# 25 Years of “Loop” Radiochemistry for PET Imaging

**DOI:** 10.1002/jlcr.70029

**Published:** 2026-04-13

**Authors:** Emily Murrell, Sahil Khan, Neil Vasdev

**Affiliations:** ^1^ Azrieli Centre for Neuro‐Radiochemistry, Brain Health Imaging Centre, Campbell Family Mental Health Research Institute, Centre for Addiction and Mental Health Toronto ON Canada; ^2^ Department of Psychiatry, Temerty Faculty of Medicine University of Toronto Toronto ON Canada

## Abstract

While conventional methods to ^11^C‐ and ^18^F‐labelled radiopharmaceuticals utilize a vial‐based approach, in the past quarter‐century, a modern, efficient, and cleaner “loop method” has been developed. This Perspective will shed light on the history behind the development of the loop radiochemistry method, the multifold advantages associated with the method over other approaches, its applications to the synthesis of ^11^C‐ and ^18^F‐labelled compounds and radiopharmaceuticals for preclinical and human PET imaging studies, its eventual expansion to diverse organic chemistry reactions and finally provide a future outlook for further developments in this field of radiochemistry.

## Graphical abstract

While conventional methods to ^11^C‐ and ^18^F‐labelled radiopharmaceuticals utilize a vial‐based approach, in the past quarter‐century, a modern, efficient, and cleaner “loop method” has been developed. The radiochemical diversity of reactions synthesized by the loop method are shown.

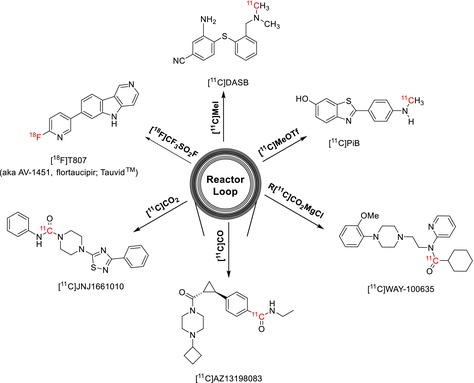

## 1 Introduction

Traditional vial‐based methods to produce positron emission tomography (PET) radiopharmaceuticals with high molar activities (A_m_) require building blocks, such as [^18^F]fluoride ion, [^11^C]iodomethane, or [^11^C]methyl triflate, to be added into a reactor vessel with precursor, solvent, and other reagents such as acids, bases, or catalysts. The reaction then is allowed to proceed in the vessel, for varying reaction times, heating, and/or stirring conditions, and the reaction mixture is then transferred onto a high‐performance liquid chromatography (HPLC) system for semi‐preparative purification followed by formulation. Owing to the short half‐lives of ^11^C (t_1/2_ = 20.3 min) and ^18^F (t_1/2_ = 109.7 min), rapid and simplified methods for radiolabelling reactions while obtaining high radiochemical yields (RCY) and achieving high radiochemical purity (RCP) and A_m_ are needed for consistent radiopharmaceutical production and applications.

The vast majority of ^11^C‐labelled radiopharmaceuticals have historically been and are still synthesized by ^11^C‐methylation reactions [1]. In the 1980s, Jewett et al. and Watkins et al. introduced the concept of “captive solvent” methods to radiolabelling wherein a radiosynthon is introduced to a solid support preloaded with precursor (and base if required), thus replacing the need of the reaction vessel [2, 3]. For *N*‐[^11^C]methylations of secondary amides, a Teflon reaction loop filled with yarn saturated with a small amount of base and precursor was described, wherein cooling to −50°C was necessary to trap the [^11^C]iodomethane onto the loop, and heating of the loop was required for the reaction to proceed [3]. In 1992, Iwata et al. described a similar extension of this concept, termed “on‐line” methylation in which a short column of a solid support was incorporated into a six‐port HPLC injection valve in place of the injection loop [4]. In this system, [^11^C]iodomethane was trapped on the reaction column prior to the addition of precursor solution.

**FIGURE 1:**
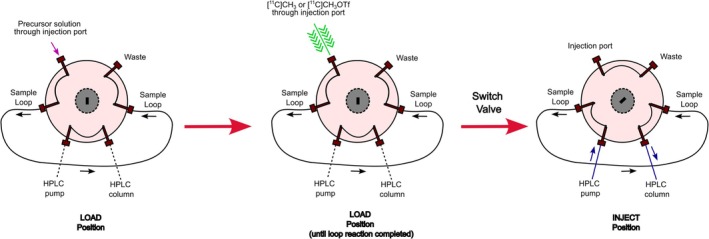
Schematic depiction of the loop reaction setup. The setup consists of loading a precursor solution to a cleaned and dried HPLC loop in the “LOAD” position through an injection port, after which the injection port is replaced by a line to deliver [^11^C]iodomethane or [^11^C]methyl triflate through the built‐in HPLC loop. This is followed by injection and HPLC purification after the reaction is completed.

In 2000, Wilson et al. reported a simplified and versatile method for ^11^C‐methylations, commonly termed the “loop method.” [5] This iteration avoids the use of any additional solid supports and cooling or heating steps by directly trapping a slow flow of [^11^C]iodomethane or [^11^C]methyl triflate (approximately 8 mL/min) in a 2‐mL stainless steel HPLC loop precoated with a small volume (80 μL—this volume of solvent used is deemed critical to evenly coat the interior of the HPLC loop) of precursor solution (with or without base), followed by an injection of the loop contents into the HPLC column system for purification (Figure 1). These reaction conditions have proven to result in high (> 90%) trapping efficiency of the [^11^C]iodomethane at ambient temperature, as well as high radiochemical yields due to the increased precursor concentration and surface area, compared with traditional vial‐based methods. Under these conditions, the elimination of additional solid supports also prevents any retention of reaction mixture in the loop, which can lead to poor resolution and less efficient HPLC separations. This iteration also generally avoids heating or cooling steps, albeit the loop method has since been adapted to utilize heat. This method was demonstrated to be amenable for multiple ^11^C‐labelled radiopharmaceuticals such as [^11^C]RTI‐32, [^11^C]Raclopride, [^11^C]Rolipram, and [^11^C]DASB.

Overall, there are multiple advantages for carrying out ^11^C‐methylation reactions in the loop method, such as (i) avoiding the use of disposable septa, vials, needles, transfer lines, columns and trapping solid supports, and so on which may interfere with radiochemical reactions; (ii) less risk of transfer losses of solution and activity when compared to vial‐based methods; (iii) reduction of cleaning procedures between radiosynthetic productions; (iv) shorter radiosynthesis times; (v) lower amounts of precursor (≤ 1 mg) that are required for limited precursors to achieve high reaction concentrations and may also facilitate purification as well as and lead to higher molar activities; and (vi) ability to label at ambient temperature is beneficial when precursors or products are not thermally stable and because mild conditions typically generate less byproducts.

## 2 Applications

In an attempt to broadly utilize the loop method, a commercial methylation apparatus was developed and patented specifically for captive solvent ^11^C‐methylation reactions [5] and was marketed as the AutoLoop by BioScan in the early 2000s. However, it was soon realized that commercial modules used for producing ^18^F‐radiotracers could be adapted to produce ^11^C‐radiotracers by the loop method. Our laboratories successfully synthesized multiple ^11^C‐labelled radiopharmaceuticals by modifying a GE TRACERlab FX_FN_ module [6], and this has since been further adapted to other commercial radiofluorination or carbon‐11 synthesis modules.

Since 2000, ^11^C‐radiopharmaceuticals have been routinely produced using in‐loop ^11^C‐methylation methodology for clinical PET research at our Brain Health Imaging Centre at the Centre for Addiction and Mental Health and multiple sites around the world (see Table 1 for a representative list of ^11^C radiopharmaceuticals). It is noteworthy that the synthesis of [^11^C]PiB using [^11^C]CH_3_OTf simplified the previous two‐step reaction for its synthesis [7].

**TABLE 1:** Highlighted radiopharmaceuticals used in clinical imaging synthesized by the loop method.

Tracer name	Biological target	Chemical structure
[^11^C]PIB	Amyloid plaques	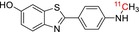
[^11^C]DTBZ	Vesicular monoamine transporter	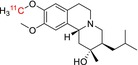
[^11^C]raclopride	Dopamine receptor D_2_	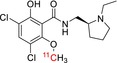
[^11^C]NOP‐1A	Nociception opioid peptide receptor	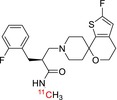
[^11^C]loperamide	p‐Glycoprotein	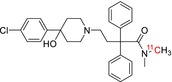
[^11^C]GSK‐189254	Histamine H_3_ receptor	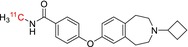
[^11^C]DASB	Serotonin transporter	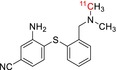
[^11^C]PE2I	Dopamine transporter	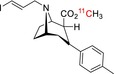
[^11^C]harmine	Monoamine oxidase A	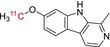
[^11^C]MeNER	Norepinephrine transporter	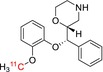
[^11^C]rolipram	Phosphodiesterase 4	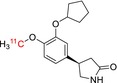
[^11^C]SCH‐23390	Dopamine receptor D_1_	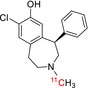
[^11^C]SB207145	Serotonin 5‐HT_4_ receptors	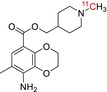
[^11^C]SKF‐82957	Dopamine receptor D_1_	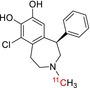
[^11^C]SB‐13	Amyloid plaques	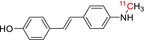
[^11^C]FLB‐457	Dopamine receptor D_2_	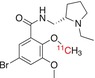
[^11^C]GSK‐215083	Serotonin 5‐HT_4_ receptor	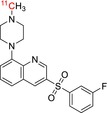
[^11^C]McN 5652	Serotonin transporter	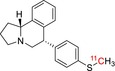
[^11^C]DAPP	Serotonin transporter	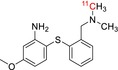
[^11^C]CPC‐222	Serotonin 5‐HT_1A_ receptor	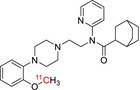
[^11^C]CUMI‐101	Serotonin 5‐HT_1A_ receptor	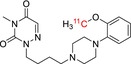
[^11^C]NNC‐112	Dopamine receptor D_1_	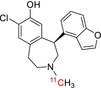
[^11^C]P943	Serotonin 5‐HT_1B_ receptor	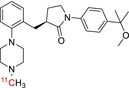
[^11^C]OMAR	Cannabinoid receptor 1	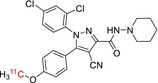

## 3 Radiochemical Diversity of the Loop Method

**FIGURE 2:**
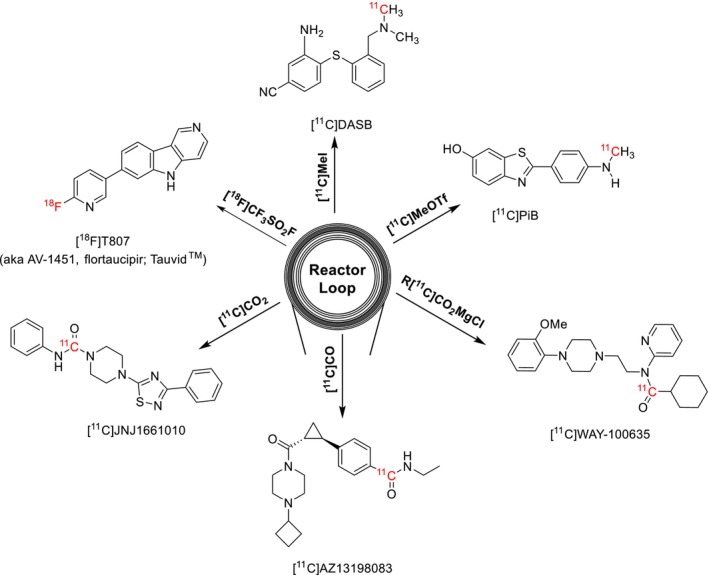
Chemical diversity of reactions synthesized by the loop method.

The loop method has recently been expanded from ^11^C‐methylation reactions to other common radiolabelling reactions and now includes loop‐based ^11^C‐acylations, [^11^C]CO_2_‐fixation, [^11^C]CO‐carbonylation, and even ^18^F‐fluorination methods (Figure 2).

Carbonyl groups are central to bioactive and drug molecules in functional groups such as amides, carboxylic acids, esters, aldehydes and ketones. One method of introducing ^11^C‐carbonyl containing groups involves the use of Grignard reagents to conduct ^11^C‐acylations. In 2007, Wadsak et al. reported the development of an automated two‐step loop‐based ^11^C‐carboxylation method used to produce [^11^C]WAY‐100635 [8]. This method involved trapping cyclotron produced [^11^C]CO_2_ onto a loop precoated with the relevant Grignard reagent, followed by converting the reaction intermediate in‐loop into its corresponding ^11^C‐acyl chloride using thionyl chloride and ultimately transferring the contents out of the loop into a reactor vessel that could be heated with a precursor molecule to achieve ^11^C‐acylation prior to HPLC purification. The same group later synthesized [^11^C]‐(+)‐PHNO by expanding the above loop method to add a reduction of the amide once the ^11^C‐acylation had taken place [9]. Both radiotracers were produced with high RCY and A_m_.

An attractive way of ^11^C‐labelling carbonyl groups is by directly utilizing the [^11^C]CO_2_ produced in‐target. In contrast to vial‐based methods, the greater reproducibility, efficiency, and higher surface to volume ratio of loop‐based methods are advantageous for [^11^C]CO_2_‐fixation reactions. In 2018, our laboratories reported the development of an in‐loop [^11^C]CO_2_ fixation reaction for synthesizing ^11^C‐radiotracers containing carbamate or urea moieties [10]. Most traditional vial‐based [^11^C]CO_2_‐fixation reactions utilize Grignard or organolithium reagents, but the introduction of reversible [^11^C]CO_2_ trapping in solution with strong organic bases presented an opportunity for loop‐based [^11^C]CO_2_‐fixation reactions. This loop method involves trapping [^11^C]CO_2_ into a reactor loop precoated with a solution of precursor and a CO_2_‐fixating base (such as DBU or BEMP) in a small volume of solvent [10]. This reaction proceeds at ambient temperature and pressure, and ^11^C‐labelled ureas and carbamates were isolated with high radiochemical purities and molar activities.

Carbonylation radiolabelling methods using [^11^C]CO enable more diverse radiochemistry including the synthesis of ^11^C‐amides. The recent commercialization of automated synthesis modules that convert [^11^C]CO_2_ to [^11^C]CO through a one‐step reduction of [^11^C]CO_2_ over molybdenum or activated charcoal columns at high temperatures (ScanSys Tracermaker) enables wider use of this technology. Traditional radiolabelling methods generally suffer from low reactivity owing to the poor solubility of [^11^C]CO in organic solvents and low concentration of [^11^C]CO. While there are solutions to make [^11^C]CO more reactive such as conducting [^11^C]CO‐carbonylations in a micro‐autoclave at high pressures or using chemical carriers that complex with CO such as diboranes and copper scorpionates, [^11^C]CO‐carbonylation in‐loop is a more versatile way to overcome these limitations. In 2019, Ferrat et al. were successful in optimizing the Pd‐Xantphos ^11^C‐aminocarbonylation reaction as a one‐step, in‐loop reaction, achieving high trapping efficiencies and radiochemical yields [11]. This reaction capability extended to labelling of diverse functional groups (^11^C‐labelled lactones, esters, and carboxylic acids) and different catalytic systems to generalize this loop method beyond aminocarbonylations. Similarly, Donnelly et al. reported the synthesis of ^11^C‐labelled drug candidates containing ^11^C‐acrylamide moieties through an in‐loop approach [12]. Both groups were able to successfully automate the radiosynthesis on commercially available radiochemistry modules. More recently, Kaur et al. developed a two‐step method for in‐loop [^11^C]CO‐carbonylations [13]. This involves [^11^C]CO‐carbonylation of an aryl halide precursor molecule in‐loop through a Pd‐Xantphos catalytic system followed by transferring the reaction mixture into a reactor preloaded with an appropriate nucleophile (OH^‐^ or OR^‐^) to form carboxylic acid and ester containing radiotracers. The two‐step approach led to higher radiochemical conversions for [^11^C]CO‐carbonylations when compared with previous one‐step loop‐based methods.

With the advantages of loop radiochemistry for developing ^11^C‐radiotracers over vial‐based methods, researchers have also sought to introduce fluorine‐18 into bioactive molecules through captive solvent methods. In 2019, our laboratories reported the first in‐loop ^18^F‐fluorination utilizing [^18^F]triflyl fluoride as the fluorine‐18 source [14]. In‐loop ^18^F‐fluorination first requires the conversion of aqueous [^18^F]fluoride ion to [^18^F]triflyl fluoride, which is then distilled into and trapped in a HPLC loop precoated with the precursor molecule, base, and aprotic solvent. In this proof‐of‐concept reaction, heating of the loop by submersion in an oil bath was also required for the radiolabelling reaction to proceed efficiently. This method was successfully applied to the synthesis of [^18^F]1‐fluoro‐4‐nitrobenzene and two ^18^F‐radiotracers ([^18^F]Tauvid and [^18^F]FEPPA) which were all produced with high RCY and A_m_ [14]. Significantly better trapping efficiencies of [^18^F]triflyl fluoride were observed for in‐loop methods when compared to vial‐based methods, attributed to the higher surface‐to‐volume ratio seen in loop radiochemistry.

## 4 Conclusions

The loop method has had a significant impact on carbon‐11 chemistry over the past 25 years and will continue to be a longstanding method of choice for production of ^11^C‐labelled compounds and radiopharmaceuticals. The methodology has proven to be reliable and simple for reactions with [^11^C]CH_3_I or [^11^C]CH_3_OTf. While applications with [^11^C]CO_2_, [^11^C]CO and ^18^F‐fluorination have been explored, there is still plenty of opportunity to expand the application of loop radiochemistry with other radionuclides and to diversify its substrate scope. Advances in [^11^C]CO_2_ fixation and commercialized modules for automated production of [^11^C]CO, as well as ^11^C‐cassette‐based modules, represent some of the numerous opportunities to expand the loop method in radiotracer production.

## Conflicts of Interest

The authors declare no conflicts of interest.

## Data Availability

Data sharing is not applicable to this article as no datasets were generated or analyzed during the current study.
